# Estimation of population affinity using cranial measurements acquired in multidetector computed tomography images of Japanese and Malay individuals

**DOI:** 10.1007/s00414-024-03386-x

**Published:** 2024-12-04

**Authors:** Suguru Torimitsu, Akari Nakazawa, Ambika Flavel, Hirotaro Iwase, Yohsuke Makino, Salina Hisham, Daniel Franklin

**Affiliations:** 1 Centre for Forensic Anthropology, School of Social Sciences, University of Western Australia, Crawley, WA 6009 Australia; 2 Department of Forensic Medicine, Graduate School of Medicine, The University of Tokyo, Tokyo, 113 − 0033 Japan; 3 Education and Research Center of Legal Medicine, Graduate School of Medicine, Chiba University, Chiba, 260–8670 Japan; 4 Department of Obstetrics and Gynecology, Graduate School of Medicine, The University of Tokyo, Tokyo, 113–8655 Japan; 5 Department of Forensic Medicine, Hospital Sultan Idris Shah Serdang, Ministry of Health Malaysia, Kajang, 43000 Malaysia

**Keywords:** Forensic anthropology, Multidetector computed tomography, Skull, Japanese, Malays, Ancestry assessment

## Abstract

It is imperative in a forensic investigation to determine the identity of an unidentified corpse, for which a crucial starting point is to establish population affinity as part of the biological profile supplied by the forensic anthropologist. The present study investigates the feasibility of using multidetector computed tomography (MDCT) images to quantify craniometric variation between Japanese and Malay populations relative to the estimation of population affinity in a forensic context. The Japanese and Malay samples comprise MDCT scans of 252 (122 female; 130 male) and 182 (84 female; 98 male) adult individuals, respectively. A total of 18 measurements were acquired, and two machine learning methods (random forest modeling, RFM; support vector machine, SVM) were applied to classify population affinity. The accuracy of the two-way pooled-sex model was 88.0% for RFM and 94.5% for SVM, respectively. The four-way population and sex model produced an overall classification accuracy of 81.3% for RFM and 91.7% for SVM. The sex-specific models of population affinity showed correct rates of classification of more than 90% in both females (90.8% for RFM and 97.6% for SVM) and males (91.2% for RFM and 97.4% for SVM). Our findings clearly indicate that the cranial measurements acquired in MDCT images can be used for the forensic classification of Japanese and Malay individuals and thus serve as a reference for forensic anthropologists attempting to identify unidentified remains.

**Supplementary Information:**

The online version contains supplementary material available at 10.1007/s00414-024-03386-x.

## Introduction

The determination of the identity of an unidentified corpse is a fundamentally important task in forensic investigations, especially in the case of dismembered, burned, severely mutilated bodies, and/or skeletal remains [1]. The first step in such an identification is DNA, dental, and fingerprint analysis. However, when a victim cannot be identified through either method, other approaches towards ascertaining identification are required [2]. The biological profile of unidentified human remains (e.g., biological sex, age, stature, and population affinity) should be estimated with a focus on general characteristics of bone morphology (e.g., shape and size– form) [3]. While the estimation of population affinity is a particularly complex topic [4], especially in modern society, forensic practitioners still need to estimate population affinity as of the biological profile [5–7]. This is important to not only assign likely ancestry, but to facilitate application of population-specific standards (where available) to estimate other biological attributes (e.g., biological sex, age, and stature).

In general, as a result of variances in skull morphology (e.g., shape and size) across different population groups, methods based on skull measurements are considered the most reliable for forensic identification with respect to population affinity [4, 8–15]. Two main methodological approaches are typically used for anthropological assessments: morphoscopic (visual or nonmetric) and morphometric (linear measurements and angles). Cranial measurements, in combination with genetic data, have been used by researchers to identify diversity across world populations and to achieve a deeper understanding of evolutionary events. It is known that functional regions of the cranium may express population affinity differently relative to admixture over time [16, 17]. Further, the incorporation of cranial measurement data in population affinity estimation improves accuracy over nonmetric characteristics and cranial morphology [15, 18].

Previous research has demonstrated that cranial morphology in Asians populations varies widely by region [19–31]. Although many academic studies have provided population affinity estimation for Western populations, few have compared cranial data of Asian skulls [15]. In addition, craniometric data used to estimate population affinity show that Hispanic skulls from the Southwestern United States are often misclassified as Asian, in particular as Japanese [32]. In other words, Hispanic and Japanese skulls share some degree of morphological similarity. To minimize the possibility of misclassification, it is important to investigate global populations relative to those of Japanese origin.

While craniometric data are available to distinguish between Japanese and Thai [15], and between Japanese and Filipino [33] skulls, no research has hitherto compared the morphologies of Japanese and Malay skulls. In 1941, during World War II, the Japanese military began Malayan campaign, and occupied the Malay Peninsula from 1942 to 1945 [34]. In addition, although less than 10,000 Japanese were living in Malaysia in 2010, this population exceeded 30,000 in 2020 [35]. There may, therefore, be some population admixture between the two groups. Accordingly, the present study investigates craniometric variation between Japanese and Malay populations, and the feasibility of estimating population affinity using data acquired in multidetector computed tomography (MDCT) images.

## Materials and methods

### Materials

The study protocol was approved by the ethics committee of the University of Tokyo (2121264NI) and the University of Western Australia (2020/ET000038).

#### Japanese population

The Japanese sample comprises postmortem CT (PMCT) scans of 252 adult corpses of known age and sex (122 females; 130 males) obtained from the Department of Forensic Medicine at the University of Tokyo between July 2017 and June 2023. The female and male samples were 19–78 (mean: 48.0 ± 17.8) years, and 19–80 (mean: 46.3 ± 18.0) years of age at death, respectively. The estimated postmortem interval for all subjects was < 14 days. The exclusion criteria included skull fractures, lethal head trauma, burn injuries, and acquired or congenital abnormalities that affected normal morphology and/or the ability to acquire reliable measurement data.

#### Malay population

The Malay sample includes MDCT scans of 182 adult living individuals (84 females; 98 males) obtained from the Hospital Kuala Lumpur for clinical cranial evaluation between July 2010 and August 2013. The female and male samples were 19–64 (mean: 34.8 ± 12.6) years, and 18–65 (mean: 37.5 ± 12.0) years, respectively. The scan images were anonymized (providing only sex, age, and population affinity as background information retained) before receipt. The entire sample was Malay (a major ethnic group in Malaysia) according to the medical records. Exclusion criteria are the same as described for the Japanese population (see above).

## Methods

For the Japanese subjects, PMCT scanning was performed using a 16-row detector CT system (Eclos; Fujifilm Co., Ltd., Tokyo, Japan). The scanning protocol was as follows: collimation of 0.625 mm, reconstruction interval of 0.625 mm, tube voltage of 120 kV, and tube current of 200 mA. For the Malay subjects, cranial imaging was performed using a 16-slice CT scanner (SOMATOM Emotion; Siemens Pte Ltd., Singapore), with an average slice thickness of 0.99 mm, tube voltage of 135 kV, and tube current of 200 mA. The images were reconstructed to the same thickness as scanning protocol. The image data, reconstructed with a soft tissue kernel, were processed on a workstation (Synapse Vincent; Fujifilm Medical Co., Ltd., Tokyo, Japan) to obtain orthogonal multiplanar reconstruction images and volume-rendered images. A three-dimensional (3D) CT image that was reconstructed using extracted bone data based on CT values was used for assessment.

**Fig. 1:**
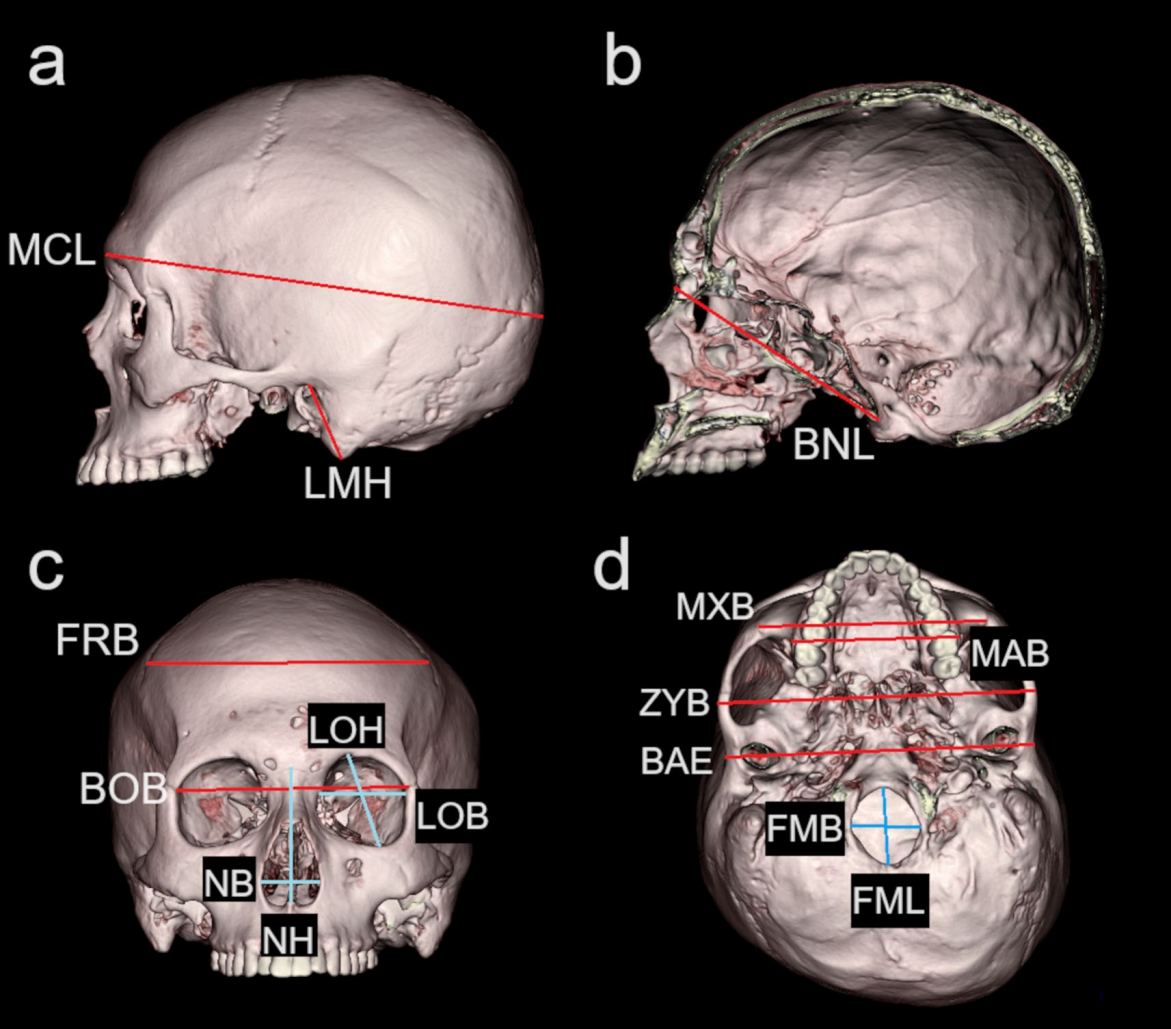
Three-dimensional computed tomography images presenting cranial measurements (see Online Resource 2 for their definitions): (**a**) maximum cranial length (MCL) and left mastoid height (LMH); (**b**) basion–nasion length (BNL); (**c**) frontal breadth (FRB), biorbital breadth (BOB), left orbit height (LOH), left orbit breadth (LOB), nasal height (NH), and nasal breadth (NB); and (**d**) bimaxillary breadth (MXB), maxillo-alveolar breadth (MAB), bizygomatic breadth (ZYB), biauricular breadth (BAE), foramen magnum length (FML), and foramen magnum breadth (FMB). Right mastoid height (RMH), right orbit height (ROH), and right orbit breadth (ROB) are not shown because they are left symmetrical

In accordance with previous research [8, 36–41] 35 cranial landmarks (Online Resource 1) were acquired in the MDCT image of each individual; these were then used to calculate 18 measurements, accurate to the nearest 0.1 mm (Fig. 1 and Online Resource 2). A subset of six subjects (three female and three male) were randomly selected; ST recollected the subset data for the assessment of intra-observer error; AN collected the subset data to assess inter-observer error. All 18 cranial measurements were acquired six times in each of the six subjects. To minimize observer recall, repeat data were acquired after a minimum two-day interval and the order of the measurements was varied each time. Measurement error was statistically quantified using the relative technical error of measurement (rTEM, %) and the coefficient of reliability (R). The acceptable rTEM range, as outlined by established anthropological research [42–44], was < 5%; R values of > 0.75 were considered sufficiently precise [38, 45]. Descriptive statistics, including range, mean, and standard deviation, were calculated to provide an overview of the sample. The Kruskal–Wallis test was used to compare measurements for the four groups (Japanese and Malay females and males); *p* < 0.05 was considered to indicate statistical significance. A series of post-hoc Mann–Whitney U tests were performed for between-group comparisons, with Bonferroni correction using the Kruskal–Wallis test; *p* < 0.0083 was considered to indicate statistical significance. The analyses were performed using Excel (Microsoft Office 2019, Microsoft, Redmond, Washington, USA).

Following previous research [46–48] that investigated the feasibility of population affinity estimation, two methods of machine learning (random forest modeling, RFM; support vector machine, SVM) were used to classify population affinity. RFM is a process of iteratively testing randomly selected samples of the original training data (bootstraps), iterating the process of refining the model in multiple trees, and aggregating the models trained in each bootstrap (bagging) [49, 50]; SVM uses data at the edge of multivariate space (the intersection of two groups) to generate classification rules by maximizing the margin between the two groups [51, 52]. The utility of the machine learning models was assessed across three scenarios: (i) a two-way model that distinguishes by population affinity (pooled-sex); (ii) a four-way model that distinguishes by population affinity and sex simultaneously; and (iii) two-way models distinguishing sex-specific (female and male) populations. Random forest feature importance was calculated in the RFM analyses. The performance of the machine learning was analyzed using R 4.4.1 (R Foundation for Statistical Computing, Vienna, Austria) with the packages “randomForest” and “e1071” [53, 54].

## Results

In the calculation of inter- and intra-observer error, rTEM and the R values ranged from 0.41 to 2.83%, and from 0.763 to 0.988, respectively (Table 1). The range, mean and standard deviation of the 18 measurements are shown in Table 2. For both the Japanese and Malay males, all mean measurement values were larger than those of females. For the same sexes, with the exception of frontal breadth (FRB) and maxillo-alveolar breadth (MAB) for males and females, and bimaxillary breadth (MXB) for males, the mean measurement values were larger in the Japanese compared to the Malay population. The Kruskal–Wallis test exhibited significant differences in all measurements between the four groups (*p* < 0.001). The results of the post-hoc tests comparing the measurements for each two groups are provided in Online Resource 3.

**Table 1:** Relative technical error of measurements (rTEM) and coefficient of reliability (*R*)

Measurement	Intra-observer error		Inter-observer error	
	rTEM	*R*	rTEM	*R*
MCL	0.41	0.988	0.68	0.965
BNL	0.64	0.978	1.21	0.932
FRB	0.92	0.952	1.66	0.827
ZYB	0.58	0.978	0.69	0.967
FML	1.08	0.918	1.32	0.895
FMB	1.01	0.929	1.38	0.834
LMH	2.50	0.842	2.70	0.816
RMH	2.76	0.799	2.83	0.763
NH	1.27	0.891	1.42	0.852
NB	1.55	0.984	2.07	0.970
LOH	1.82	0.781	2.75	0.782
ROH	1.89	0.877	2.77	0.789
LOB	1.85	0.924	2.26	0.862
ROB	1.58	0.936	2.08	0.854
MXB	1.25	0.927	1.72	0.884
MAB	1.34	0.960	1.56	0.936
BOB	0.64	0.981	0.67	0.979
BAE	1.20	0.947	1.49	0.899

**Table 2:** Descriptive statistics of 18 cranial measurements

	Japanese				Malay			
	Female (*n* = 122)		Male (*n* = 130)		Female (*n* = 84)		Male (*n* = 98)	
Measurement	Range	Mean ± SD^a^	Range	Mean ± SD	Range	Mean ± SD	Range	Mean ± SD
MCL (mm)	158.7–192.7	171.9 ± 5.8	170.3–201.0	183.7 ± 6.0	153.7–183.0	169.1 ± 6.2	165.2–200.0	179.4 ± 6.9
BNL (mm)	89.9–107.4	99.6 ± 3.5	94.6–124.4	106.3 ± 4.1	85.1–105.5	95.6 ± 4.2	94.6–114.2	102.9 ± 3.9
FRB (mm)	85.4–120.4	105.2 ± 6.9	93.9–127.1	107.2 ± 6.4	96.8–128.3	111.1 ± 7.0	99.5–132.7	116.3 ± 6.6
ZYB (mm)	123.0–139.0	130.8 ± 3.9	124.9–150.8	139.6 ± 4.9	112.6–141.1	126.6 ± 5.3	120.0–145.6	134.1 ± 5.3
FML (mm)	29.7–40.4	34.8 ± 2.2	31.2–42.0	36.6 ± 2.3	27.1–39.1	32.9 ± 2.3	29.5–42.0	34.5 ± 2.4
FMB (mm)	24.6–33.6	29.1 ± 1.9	26.6–36.1	30.5 ± 1.7	21.4–34.1	27.9 ± 2.5	24.5–34.3	29.1 ± 2.2
LMH (mm)	21.2–36.1	29.7 ± 3.0	27.3–42.1	34.1 ± 2.9	20.0–33.8	27.5 ± 2.7	24.7–38.6	31.9 ± 2.9
RMH (mm)	22.1–36.9	29.5 ± 3.1	28.3–42.9	34.5 ± 3.0	19.9–35.3	28.0 ± 2.8	25.8–38.3	32.4 ± 3.0
NH (mm)	42.7–62.3	52.5 ± 3.3	48.1–62.5	55.7 ± 2.9	37.6–55.8	48.0 ± 3.1	47.8–58.2	52.4 ± 2.6
NB (mm)	20.8–32.8	25.7 ± 2.3	22.2–31.4	26.7 ± 2.0	20.2–29.3	24.2 ± 1.7	18.8–29.5	25.0 ± 2.2
NOH (mm)	36.5–45.7	40.7 ± 2.1	37.0–47.5	41.8 ± 2.0	31.7–41.6	37.4 ± 1.8	33.1–42.4	38.3 ± 2.1
ROH (mm)	35.3–45.3	40.3 ± 2.2	35.0–46.2	40.9 ± 2.2	32.0–41.6	36.6 ± 1.9	32.5–42.6	37.6 ± 2.3
LOB (mm)	34.9–43.3	37.9 ± 1.6	35.0–44.1	39.3 ± 1.6	33.1–39.8	36.0 ± 1.5	33.7–41.3	37.3 ± 1.7
ROB (mm)	35.3–42.3	37.7 ± 1.4	35.7–44.0	39.2 ± 1.5	32.7–40.1	36.5 ± 1.6	33.5–41.4	38.0 ± 1.7
MXB (mm)	83.4–103.1	95.1 ± 3.9	84.7–108.4	98.6 ± 5.1	86.5–102.4	94.6 ± 3.8	89.6–109.5	100.2 ± 4.5
MAB (mm)	50.5–70.7	61.1 ± 3.6	52.9–75.4	65.9 ± 4.1	56.2–71.2	62.5 ± 3.5	56.7–74.9	66.1 ± 3.8
BOB (mm)	88.1–103.5	95.8 ± 2.9	93.0–109.6	100.6 ± 3.7	88.2–103.6	95.6 ± 3.4	88.2–107.5	99.8 ± 4.1
BAE (mm)	112.8–130.1	120.4 ± 4.1	113.2–137.6	127.5 ± 4.9	105.1–131.1	117.7 ± 5.1	108.4–130.7	122.4 ± 5.3

^a^ Standard deviation

The results of machine learning models are summarized in Tables 3, 4, 5 and 6. The accuracy of the two-way pooled-sex model was 88.0% for RFM and 94.5% for SVM (Table 3). Higher classification accuracy was achieved for the Japanese individuals. The four-way model demonstrated an overall classification accuracy of 81.3% for RFM and 91.7% for SVM (Table 4). Japanese males had the highest rates of correct classification, and Malay males the lowest. As shown in Tables 5 and 6, sex-specific population affinity analyses resulted in classification rates above 90% in both females (90.8% for RFM and 97.6% for SVM) and males (91.2% for RFM and 97.4% for SVM).

**Table 3:** Classification matrix showing classification of groups according to population affinity

Group	RFM			SVM		
	JP	MA	% Correct	JP	MA	% Correct
JP	227	25	90.1%	240	12	95.2%
MA	27	155	85.2%	12	170	93.4%
All			88.0%			94.5%

RFM, random forest modeling; SVM, support vector machine; JP, Japanese; MA, Malay

**Table 4:** Classification matrix showing classification of groups according to population affinity and sex

Group	RFM					SVM				
	JPF	JPM	MAF	MAM	% Correct	JPF	JPM	MAF	MAM	% Correct
JPF	101	9	6	6	82.8%	113	6	0	3	92.6%
JPM	9	115	0	6	88.5%	3	125	0	2	96.2%
MAF	8	0	69	7	82.1%	4	0	75	5	89.3%
MAM	15	6	9	68	69.4%	5	4	4	85	86.7%
All					81.3%					91.7%

JPF, Japanese female; JPM, Japanese male; MAF, Malay female, MAM, Malay male

**Table 5:** Classification matrix showing classification of groups according to sex-specific population affinity (female)

Group	RFM			SVM		
	JPF	MAF	% Correct	JPF	MAF	% Correct
JPF	115	7	94.3%	122	0	100%
MAF	12	72	85.7%	5	79	94.0%
All			90.8%			97.6%

RFM, random forest modeling; SVM, support vector machine; JPF, Japanese female; MAF, Malay female

**Table 6:** Classification matrix showing classification of groups according to sex-specific population affinity (male)

Group	RFM			SVM		
	JPM	WAM	% Correct	JPM	MAM	% Correct
JPM	120	10	92.3%	128	2	98.5%
MAM	10	88	89.8%	4	94	95.9%
All			91.2%			97.4%

RFM, random forest modeling; SVM, support vector machine; JPM, Japanese male; MAM, Malay male

**Fig. 2:**
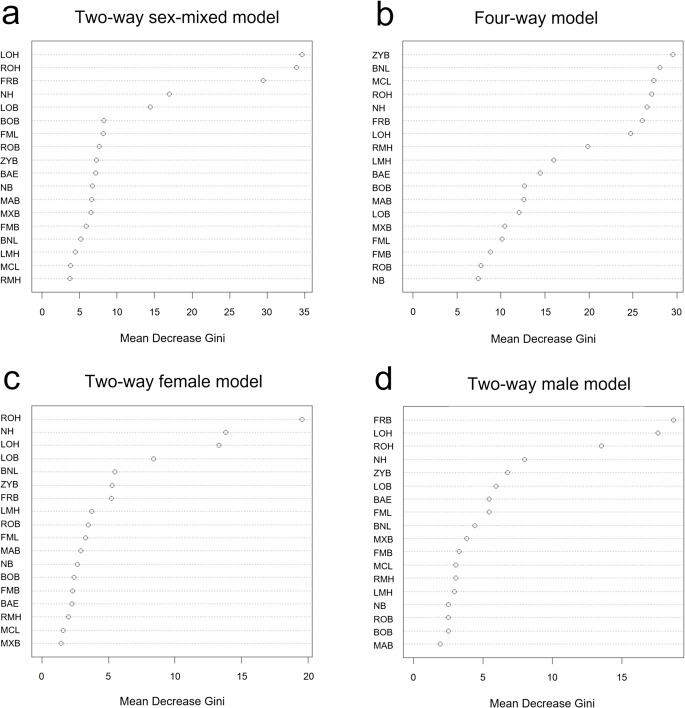
Random forest feature importance (Mean Decrease Gini) for the response variable. Mean Decrease Gini indicates a decrease in the Gini coefficient after variable substitution. A higher value indicates a higher importance. (**a**) two-way sex-mixed model, (**b**) four-way population affinity and sex model, (**c**) two-way female model, and (**d**) two-way male model

The random forest feature importance showed that left orbit height (LOH), right orbit height (ROH), and nasal height (NH) had strong weighted measurements relative to correct classification in the two-way models; bizygomatic breadth (ZYB), basion–nasion length (BNL), and maximum cranial length (MCL) are the strong weighted measurements in the four-way model (Fig. 2).

## Discussion

In the present study intra- and inter-observer errors were small and were likely negligible. Taking this into account, the cranial measurements acquired using 3D CT images in this study are highly reliable and reproducible.

It is widely acknowledged that sexual dimorphism in cranial size and shape is common among geographically and genetically distant populations [36]. While significant research suggests that Asians present a smaller degree of cranial sexual dimorphism compared to than other populations [55, 56], there is a considerable body of published research that shows quantifiable dimorphism in numerous Asian populations (Thai, Indonesian, and Filipino) [11, 15, 33, 57]. The latter accords with the outcomes of our analyses of Japanese and Malay individuals.

The size and shape of the skull also exhibits significant variation among populations [58–60]. Asian skulls tend to be shorter and broader than those of other global populations [61, 62]. In addition, craniometric studies show significant differences among Asian populations [19, 20]. In particular, the North/East (Sinodonty) and Southeast (Sundadonty) Asian groups can be distinguished based on their cranial morphology [19, 21–29]. In addition, this study found differences between Japanese and Malay individuals in most cranial measurements. In addition, MCL in the Japanese sample was larger than a Thai (females, 166.85 ± 7.76 mm; males, 174.25 ± 6.52 mm) [11] and Korean population (females, 165.0 ± 6.6 mm; males, 174.0 ± 7.7 mm) [63], which is consistent with previous reports [15, 64]. The NH in the Japanese sample of this study is larger than that in a Filipino population (females, 48.07 ± 2.96 mm; males, 51.24 ± 2.48 mm), while the nasal breadth (NB) in the Japanese sample was smaller than the Filipinos (females, 26.36 ± 1.78 mm; males, 27.49 ± 1.82 mm) [33]. Both the NH and NB in a Thai population reported by Kongkasuriyachai et al. [15] were larger than those in the Japanese sample examined in the present study. Such differences of skull characteristics may reflect a complex Asian population that has regional differences [15, 65].

The results of this study show that the rates of correct classification for Japanese and Malays using the pooled-sex model were over 85%, and even where sex was classified simultaneously, the rates exceeded 80%. This indicates that cranial measurements derived from MDCT images can be used to classify Japanese and Malays individuals to their respective population of origin with a high degree of expected accuracy. The present study also showed that accuracy was higher in Japanese compared to Malay skulls. This reflects greater variability in the Malay relative to the Japanese skulls [15]. Hisham and Ibrahim [66] used discriminant analyses to classify different ethnic groups in Malaysian populations to distinguish Malays, Chinese, and Indians, demonstrating a correct classification rate of 69.57%. In addition, Hisham and Ibrahim [67] reported that when Malaysian skulls were tested using Fordisc 3, only 32.33% of the sample were correctly classified as Asian. These results also showed a potential mixed population affinity for the Malaysian population. This is not surprising given it is known that people in the Malay Peninsula comprise various subethnic groups that have different ancestral origins based on centuries-past migrations [68]. However, no human skeletal collections from Malaysia with documented parameters are currently available [66]. On the other hand, both clinical and PMCT data of Malays are useful in forensic anthropological research.

In this study, correct classification by population affinity using the sex-specific models exceeded 90%. Our previous study also demonstrated that the estimation of population affinity by sex yielded higher classification rates [46]. These results indicate that an accurate sex estimation is an important a priori consideration in estimating population affinity. In other words, it is hypothesized that if unidentified skulls can be presumed to be female or male, population affinity can be more accurately estimated using sex-specific models. However, few studies have been conducted on sex-specific population affinity estimation with the use of skulls, and further research is warranted.

Previous research has indicated that MCL and ZYB are important variables in population affinity estimation [46, 50, 69]. This study also found that MCL and ZYB were important factors in the four-way model, suggesting that these measurements can be used for population affinity classification in various global populations. However, LOH, ROH, and NH were important variables in distinguishing Japanese from Malay skulls in the 2-way models. There have been no previous reports of the importance of those variables for population affinity estimation, which may be due to differences in the sample populations. It should be recognized that some populations are either understudied, or have not been examined at all, relative to the estimation of population affinity. Therefore, it is imperative that further craniometric studies of skulls from other populations be conducted to further develop and refine the various tools that are available to the forensic practitioner to assist towards the identification of an unknown decedent(s).

The mean age of the Japanese sample was higher than that of the Malays. It is important to note that previous studies have discussed the possibility of age-related changes, such as increased size of parts of the cranium in middle-aged to elderly people [70]. However, Albert et al. [71] reported a small increase (1.1 mm to 1.6 mm) in craniofacial dimensions in the elderly; therefore based on the latter observation, age is unlikely a major factor in the misclassification rate that is observed in this study.

The majority of previous craniometric studies estimating population affinities have involved the analysis of data acquired from physical specimens [11, 15, 33]. However, few studies [46, 66, 67, 72] have investigated the feasibility of estimating population affinity using MDCT scanning techniques that are similar to this study. Medical imaging technology facilitates high-resolution visualization of the skeleton, providing accurate identification in a timely and nondestructive manner to aid in developing osteometric standards and anthropological analyses [73–76]. Sharing CT data between institutions in different countries allows collection of global and contemporary multi-population data and provides an improved understanding of the diversity of cranial morphology that is related to ancestral origins, in particular for the identification of human remains in a forensic or humanitarian context.

It is important to consider that cranial features and measurements are phenotypic characteristics that are determined in part by several factors, including heritability, repeated mutations, cultural influences, climate and nutrition [77–80]. These factors could not be further examined in the present study, as all scans were anonymized prior to receipt, with the exception of sex, age, and population affinity. In addition, some studies have demonstrated secular changes in both craniometric and nonmetric traits, suggesting that changes in cranial size and shape may occur [81, 82]. In fact, in Japan, brachycephalization has been ongoing since the medieval period; changes in the shape of the cranium have been reported in many countries over the past 100 years [83]. For this reason, it is necessary to follow and investigate secular changes in cranial measurement over time.

The literature clearly indicates that the majority of forensic anthropological studies of population affinity have focused on the skull, although postcranial elements, including the femur and tibia, may also provide potentially useful information [48, 84, 85]. For this reason, further research that considers other skeletal measurements based on CT imaging is required to further investigate the feasibility of population affinity estimation using the post-cranial skeleton.

This present study had several limitations. First, the data were collected from two facilities using different detector CT systems that have different conditions for reconstructed images. While it does not seem likely that these issues would significantly affect measurements, the precise difference was unable to be quantified. Second, this study used PMCT and CT data from living patients. Although it is unlikely that the geometry and measurements would change significantly between the ante- and postmortem periods, the difference was not unable to be investigated. Finally, although this study used machine learning for analysis, there are other analytical methods available. In the future, the exploration of the advanced statistical analyses and the use of different types of data on craniofacial dimensions (e.g., geometric morphometrics and morphoscopic features) are proposed for application in larger samples to identify trends and obtain insight into population affinities.

## Conclusions

The present study demonstrated that craniofacial measurements obtained from 3D CT images provide accurate statistical classification of Japanese and Malay individuals relative to their respective population of origin. This study also demonstrated the potential contribution of baseline measurements to forensic practice, providing valuable data on population affinity in the Asian populations. These results serve as a reference for forensic anthropologists working to identify human skeletal remains. Further, MDCT data, cranial and post-cranial, should be collected from other populations to facilitate further study of more diverse population samples.

## Electronic supplementary material

Below is the link to the electronic supplementary material.

Supplementary Material 1

Supplementary Material 2

Supplementary Material 3

## Data Availability

The datasets generated during and/or analysed during the current study are available from the corresponding author on reasonable request.
